# O Risco Desconhecido de Hipercolesterolemia Familiar no Desenvolvimento de Doença Cardiovascular Aterosclerótica

**DOI:** 10.36660/abc.20210168

**Published:** 2021-04-08

**Affiliations:** 1 Universidade Federal de Pernambuco Centro de Ciências da Saúde Departamento de Nutrição RecifePE Brasil Departamento de Nutrição, Centro de Ciências da Saúde, Universidade Federal de Pernambuco, Recife, PE - Brasil.; 2 Universidade Federal da Paraíba Centro de Ciências da Saúde Departamento de Nutrição João PessoaPB Brasil Departamento de Nutrição, Centro de Ciências da Saúde, Universidade Federal da Paraíba, João Pessoa, PB - Brasil.

**Keywords:** Doenças Cardiovasculares/mortalidade, Aterosclerose, Fatores de Risco, Diagnóstico Precoce, Transtornos de Metabolismo, Hiperlipoproteinemia Tipo II

As doenças cardiovasculares (DCV) são reconhecidas como as principais causas de óbito na idade adulta no mundo.^1^^,^^2^ Diversos fatores, como tabagismo, hipertensão arterial, obesidade, síndrome metabólica e hipercolesterolemia, têm sido descritos como importantes fatores de risco no desenvolvimento de DCV e morte prematura.^3^ A hipercolesterolemia pode ser classificada como de origem primária ou secundária. A hipercolesterolemia primária é devida a defeitos geneticamente determinados no metabolismo de lipídios ou lipoproteínas; a hipercolesterolemia secundária pode ser causada por estilo de vida inadequado, doenças ou medicamentos.^3^

A hipercolesterolemia familiar (HF) é uma doença autossômica dominante caracterizada por mutações em genes na codificação de proteínas envolvidas no metabolismo de lipoproteínas de baixa densidade (LDL) que ocorrem na presença de níveis elevados de colesterol plasmático e LDL associados a sinais clínicos de xantoma tendíneo. A HF é responsável por aproximadamente 10% dos eventos cardiovasculares em indivíduos com menos de 50 anos.^4^^–^^6^ Na HF, as mutações podem estar presentes no gene do receptor de LDL, no gene que codifica a apoproteína B-100 ou no gene autossômico recessivo LDLRAP1 e na hipercolesterolemia familiar autossômica dominante (HCHOLA3), variante do gene PCSK9.^7^ Essas mutações levam ao comprometimento da interação apoproteína-receptor e resultam em colesterol plasmático elevado, níveis elevados de LDL e risco de desenvolvimento de doença aterosclerótica.^5^^,^^8^

A HF é uma das doenças monogênicas mais comuns, reconhecida pela Organização Mundial da Saúde como um problema de saúde pública mundial. Apesar de sua ampla incidência, o diagnóstico precoce da HF ainda não é amplamente realizado e, consequentemente, a adoção de tratamentos preventivos para a hipercolesterolemia é precária.^9^ Portanto, pode-se sugerir que o diagnóstico precoce da HF e o tratamento adequado são essenciais para prevenir ou pelo menos retardar o início dos eventos cardiovasculares. A triagem em cascata da FH é uma forma crucial e econômica de prevenir processos ateroscleróticos, devendo ser realizada em todos os familiares de primeiro, segundo e terceiro graus de pacientes com diagnóstico de HF.

A importância da triagem e do tratamento precoce da HF tem sido alvo de preocupação na comunidade científica. Em um manuscrito recente, Santos Filho et al.,^10^ investigaram se indivíduos brasileiros com hipercolesterolemia sabiam do risco de desenvolver DCV e se apresentavam histórico familiar.^10^

Para responder a essa questão, os autores utilizaram um banco de dados de 70.000 brasileiros que passaram por uma avaliação de saúde de rotina obrigatória entre 2006 e 2016 no Hospital Israelita Albert Einstein em São Paulo, Brasil. Desses, 1.987 indivíduos (2,8%) preencheram os critérios de inclusão para o diagnóstico de HF (idade ≥18 e colesterol LDL em jejum ≥190 mg/dL sem estatinas ou ≥160 mg/dL em tratamento com estatinas). Desses, 200 indivíduos foram convidados aleatoriamente por telefone a participar do estudo em 2017. Além das avaliações clínicas e ambulatoriais, foram investigadas questões sobre hipercolesterolemia, como conhecimento sobre HF, diagnóstico, adesão ao tratamento, triagem em cascata para HF em parentes de primeiro grau e percepção do risco de DCV.

A maioria dos participantes era do sexo masculino, 95% com ensino superior e 12% com episódio anterior de DCV (infarto do miocárdio, angina, revascularização do miocárdio ou acidente vascular cerebral). Além disso, o estudo identificou que 97% dos participantes sabiam ter níveis elevados de colesterol e, uma porcentagem significativa — 76% — relatou ter um parente de primeiro grau com colesterol alto. Porém, apenas 4,5% (n=9) dos participantes tiveram seus familiares convocados para verificação de seus níveis de colesterol sérico. Analisando os resultados acima, pode-se sugerir que a triagem familiar não parece ser realizada de forma ampla e eficaz.

Ainda em relação à percepção do quadro de hipercolesterolemia para a ocorrência de DCV, apenas 18% dos participantes reconheceram o quadro de hipertensão como fator de risco para DCV. Por outro lado, 71% dos participantes consideravam a diabetes mellitus como o fator de risco mais relevante para DCV. Esses achados denotam a pouca importância atribuída à hipercolesterolemia como fator de risco para DCV na população brasileira analisada.

Reforçando o desconhecimento da hipercolesterolemia como importante fator de risco para DCV, embora pouco mais de 2/3 dos participantes fizessem consultas regulares, apenas 1/3 relatou conhecimentos sobre o colesterol LDL recomendado. Em indivíduos com suspeita de HF, um pequeno número (24,5%) já ouviu falar de HF. A média de idade dos participantes com suspeita de diagnóstico de HF foi de 35 anos, revelando a falta de triagem precoce. Estabeleceu-se diagnóstico genético para apenas 17% dos participantes e apenas 4% tinham ouvido falar de xantoma.

O estudo representa uma contribuição relevante para a população brasileira, visto que há carência de estudos realizados no Brasil que avaliem o conhecimento dos pacientes sobre as implicações da hipercolesterolemia no desenvolvimento das DCV. Ressalta-se que os indivíduos avaliados possuíam alto nível socioeducativo, o que aumenta a importância do conhecimento e atenção à HF por parte do público em geral. Os dados do estudo demonstram que os brasileiros precisam de mais informações sobre a HF e de campanhas educacionais em massa sobre o risco da HF no desenvolvimento de DCV e na mortalidade.
