# Supplementary material for: O Risco Desconhecido de Hipercolesterolemia Familiar no Desenvolvimento de Doença Cardiovascular Aterosclerótica
Source: Arq Bras Cardiol. 2021 Apr 8;116(4):713–4. [Article in Portuguese] doi: 10.36660/abc.20210168 (PMC8121399; doi:10.36660/abc.20210168)
Supplement: Translation

# Translation: The Unknown Risk of Familial Hypercholesterolemia in the Development of Atherosclerotic Cardiovascular Disease

**Affiliations:** 1 RecifePE Brazil Departamento de Nutrição, Centro de Ciências da Saúde, Universidade Federal de Pernambuco, Recife, PE - Brazil.; 2 João PessoaPB Brazil Departamento de Nutrição, Centro de Ciências da Saúde, Universidade Federal da Paraíba, João Pessoa, PB - Brazil.

**Keywords:** Cardiovascular Diseases/mortality, Atherosclerosis, Risk Factors, Early Diagnosis, Lipid Metabolism Disorders, Hyperlipoproteinemia Type II

Cardiovascular diseases (CVD) are recognized as the main causes of death in adulthood in the world.^1^^,^^2^ A number of factors, such as smoking, arterial hypertension, obesity, metabolic syndrome and hypercholesterolemia have been described as important risk factors in the development of CVD and premature death.^3^ Hypercholesterolemia can be classified as primary or secondary in origin. Primary hypercholesterolemia is due to genetically determined defects in lipid or lipoprotein metabolism; secondary hypercholesterolemia can be caused by inadequate lifestyle, diseases or medications.^3^

Familial hypercholesterolemia (FH) is an autosomal-dominant disorder characterized by mutations in genes in the encoding of proteins involved in low-density lipoprotein (LDL) metabolism that occur in the presence of high plasma cholesterol and LDL levels associated with clinical signs of tendon xanthoma. FH is responsible for approximately 10% of cardiovascular events in people under 50 years of age.^4^^–^^6^ In FH, mutations may be present in the LDL receptor gene, in the gene-encoding Apoprotein B-100 or in autosomal recessive LDLRAP1 gene and in autosomal dominant familial hypercholesterolemia (HCHOLA3) variant of the PCSK9 gene.^7^ These mutations lead to an impairment in the apoprotein-receptor interaction and result in high plasma cholesterol, high LDL levels and risk of development of atherosclerotic disease.^5^^,^^8^

FH is one of the most common monogenic diseases, recognized by the World Health Organization as a worldwide public health problem. Despite its widespread incidence, early diagnosis of FH is still not widely performed and, consequently, the adoption of preventive treatments for hypercholesterolemia is impaired.^9^ Therefore, it is reasonable to suggest that early diagnosis of FH and appropriate treatment are essential to prevent or at least delay the onset of cardiovascular events. Cascade screening of FH is a crucial and cost-effective way to prevent atherosclerotic processes and should be performed in all of the first, second and third-degree relatives of patients diagnosed with FH.

The importance of screening and early treatment of FH has been a target of concern in the scientific community. In a recent manuscript, Santos Filho et al.^10^ investigated whether Brazilian subjects with hypercholesterolemia knew the risk of developing CVD and whether they had family history.^10^

To answer this question, the authors used a database of 70.000 Brazilian individuals who had undergone a mandatory routine health assessment between 2006 and 2016 at Hospital Israelita Albert Einstein in São Paulo, Brazil. Of those, 1,987 subjects (2.8%) met the inclusion criteria for FH diagnosis (age ≥18 and fasting LDL-c ≥190 mg/dL without statins or ≥160 mg/dL if on statin therapy). Of these, 200 individuals were randomly invited by phone to participate in the study in 2017. In addition to clinical and outpatient evaluations, questions about hypercholesterolemia, such as knowledge about FH, diagnosis, treatment adherence, cascade screening for FH in first-degree relatives and perception of CVD risk were investigated.

The majority of the participants were males, 95% had higher education and 12% had a previous event of CVD (myocardial infarction, angina, myocardial revascularization or stroke). In addition, the study identified that 97% of the participants were aware of having high cholesterol levels and, a significant percentage — 76% — reported having a first-degree relative with high cholesterol. However, only 4.5% (n=9) of the participants had their relatives called to check serum cholesterol levels. Analyzing the results above, it is reasonable to suggest that family screening does not seem to be carried out broadly and effectively.

Still regarding the perception of the hypercholesterolemia condition for the occurrence of CVD, only 18% of the participants recognized the condition of hypertension as a risk factor for CVD. On the other hand, 71% of the participants considered diabetes mellitus as the most relevant risk factor for CVD. These findings denote the minor importance given to hypercholesterolemia as a risk factor for CVD in the Brazilian population analyzed.

Reinforcing the lack of knowledge of hypercholesterolemia as an important risk factor for CVD, although a little more than 2/3 of the participants had regular consultations, only 1/3 reported knowledge about their recommended LDL-c. In individuals with suspected FH, a small number (24.5%) had heard of FH. The mean age of the participants suspected with FH diagnosis was 35, revealing the lack of early screening. Genetic diagnosis had been established for only 17% of the participants and only 4% had heard of xanthoma.

The study represents a relevant contribution to the Brazilian population as there is a lack of studies conducted in Brazil assessing the knowledge of patients about the implications of hypercholesterolemia for the development of CVD. It should be noted that the subjects evaluated had a high social and educational level, which increases the importance of knowledge and attention to FH among the general public. The study data demonstrates that Brazilians need more information about FH and mass education campaigns need to be conducted on the risk of FH in the development of CVD and mortality.
